# Decontamination of Minimally-Processed Fresh Lettuce Using Reuterin Produced by *Lactobacillus reuteri*

**DOI:** 10.3389/fmicb.2018.01421

**Published:** 2018-07-04

**Authors:** Paul T. Asare, Anna Greppi, Martina Stettler, Clarissa Schwab, Marc J. A. Stevens, Christophe Lacroix

**Affiliations:** Laboratory of Food Biotechnology, Institute of Food, Nutrition and Health, ETH Zürich, Zürich, Switzerland

**Keywords:** reuterin, biopreservation, minimal processing, *Lactobacillus reuteri*, acrolein

## Abstract

Over the last years the demand for pre-washed, fresh-cut, and minimally-processed (MP) produce has increased. MP fresh vegetable are rapidly spoiled, whereas there is consumers’ concern about chemical disinfection treatments such as with chlorine. A promising antimicrobial is reuterin, a broad-spectrum-antimicrobial compound produced by food-grade *Lactobacillus reuteri* from glycerol. In aqueous solution, reuterin is a dynamic system consisting of 3-hydroxypropionaldehyde (3-HPA), its hydrate, its dimer as well as acrolein, which was recently identified as the main antimicrobial component of the system. Here, we tested the use of reuterin containing similar 3-HPA levels but different acrolein concentrations for decontaminating and preserving fresh-cut lettuce. Crude reuterin (CR) was produced by biotransformation of 600 mM glycerol using *L. reuteri* DSM 20016T. CR preparations were further incubated for 16 h at 50°C to produce enhanced reuterin (ER) with raised concentration of acrolein. Fresh-cut iceberg lettuce (*Lactuca sativa)* was washed using CR (1.5–1.9 mM acrolein) and ER (7.2–21.9 mM acrolein) solutions at 4°C, or sodium hypochloride (250 mg/L) and tap water, and compared with unwashed lettuce. Washed lettuce samples were packed under modified atmosphere (2% O_2_, 5% CO_2_, and 93% N_2_) and stored for 13 days at 4°C. Application of ER containing 12.1, 20.9, or 21.9 mM acrolein reduced the initial viable plate counts of *Enterobacteriaceae* (by 2.1–2.8 log CFU/g), and yeasts and molds (by 1.3–2.0 log CFU/g) when compared with unwashed samples. In contrast, reuterin solutions containing 7.2 mM acrolein, sodium hypochlorite and tap water only showed very limited and transient, or no effects on the cell loads of lettuce after washing and during storage. Visual assessment of leaves washed with ER showed acrolein concentration-dependent discoloration noticeable already after 3 days of storage for the highest acrolein concentrations. Discoloration became severe for all ER treatments after 7 days, while the other treatments preserved the aspect of washed lettuce. Our data show the predominant role of acrolein as the main antimicrobial component of the reuterin system for food biopreservation. Reuterin preparations with enhanced acrolein concentration of 12.1 mM and higher were effective to reduce plate counts of *Enterobacteriaceae* and yeasts and molds washed lettuce until day 7 but induced pronounced discoloration of lettuce.

## Introduction

The market of pre-washed, fresh-cut and minimally-processed (MP) vegetable produce is rising worldwide. MP fresh vegetable produce contain complex bacterial communities which may include spoilage microbes, e.g., fluorescent *Pseudomonas* spp. and *Erwinia carotovora*, and pathogens (Ragaert et al., 2007; Barth et al., 2009). The consumption of these produce has been associated with foodborne outbreaks by *Salmonella enterica*, pathogenic *Escherichia coli, Shigella* spp., *Campylobacter* spp., *Listeria monocytogenes, Staphylococcus aureus, Yersinia* spp. and *Bacillus cereus* (Harris et al., 2003; FAO/WHO, 2008; Critzer and Doyle, 2010; Fatica and Schneider, 2011). Therefore, suppliers are increasingly interested in implementing natural antimicrobial compounds for decontaminating the produce and providing high-quality and safe products.

Post-harvest microbial contamination rather than contamination in the field is a major cause of human pathogenic bacteria on MP fresh produce (Jensen et al., 2013). Tissue damages associated with peeling, cutting and slicing of MP fresh produce reduces the defense against pathogens (Allende et al., 2004). Internalization of fresh produce by enteric pathogens through cut surfaces reduces their exposure to sanitizing agents (Erickson, 2012). Moreover, microorganisms can adhere to the surface of freshly harvested fruits and vegetables and may survive decontamination steps due to the formation of biofilms (Allende et al., 2008). The main decontaminating steps in the processing chain of MP fruits and vegetables are washing and disinfection. Washing removes soil, insects and other debris and has an added advantage of reducing microbial loads (Gil et al., 2009). However, washing water can serve as a vehicle for dispersal of microorganisms (Holvoet et al., 2012). Thus, sanitation of produce is pivotal for guaranteeing quality and safety for human consumption.

Chlorine is the most widely used washing and sanitizing agent in the processing of fruits and vegetables (FAO/WHO, 2008). However, because of the increasing public health concerns about possible formation of chlorinated organic compounds, European countries such as Germany, The Netherlands, Denmark, and Belgium, as well as Switzerland have banned the use of chlorine in fresh-cut produce (Betts and Everis, 2005). Several alternatives to chlorine such as chlorine dioxide, ozone, organic acids, peracetic acid (PAA), and hydrogen peroxide have been gaining interest in recent years but none of them were found to have the expected requirements (Ölmez and Kretzschmar, 2009). Among alternative preservation technologies, a particular attention has been paid to biopreservation, which is defined as the use of naturally-produced compounds for decontamination and preservation (Lacroix, 2011). A promising broad spectrum biopreservative agent is the bacterial metabolite reuterin, produced by *Lactobacillus reuteri* with antimicrobial activity against Gram-positive and Gram-negative bacteria, yeasts and molds (Cleusix et al., 2007; Stevens et al., 2010).

Reuterin is produced by certain strains of *L. reuteri* during the anaerobic fermentation of glycerol. In aqueous solution, reuterin is a dynamic system consisting of 3-hydroxypropional dehyde (3-HPA), its hydrate 1,1,3-propanetriol, its dimer 2-(2-hydroxyethyl)- 4-hydroxy-1,3-dioxane, and acrolein (Vollenweider and Lacroix, 2004; Engels et al., 2016). The 3-HPA can be further converted to 1,3-propanediol (1,3-PD) in the presence of glucose, but large amounts of reuterin are excreted into the medium when glucose is low (Stevens et al., 2013). Acrolein was recently shown to be the main component responsible for the antimicrobial activity of reuterin (Engels et al., 2016). 3-HPA and acrolein interconversion does not occur at 4°C and pH 4, while at higher temperatures the equilibrium is shifted toward acrolein (Engels et al., 2016). Minimal inhibitory concentrations (MIC) and bactericidal concentrations (MBC) of reuterin (determined as 3-HPA) were measured in the ranges 0.5–15 mM for *Listeria* spp., 35 mM for *Bacillus subtilis*, 0.3–50 mM for Gram-negative *E. coli* and *P. aeruginosa*, and 0.15–0.98 mM for yeasts (Stevens et al., 2010).

The aim of the present work was to investigate whether biotechnology-produced reuterin can be used for decontamination of fresh lettuce, and the role of acrolein as the main antimicrobial compound of reuterin in a food system. The washing efficacy of crude reuterin (CR) and reuterin with enhanced acrolein concentrations (ER) was tested in reducing the microbial population of MP fresh-cut lettuce during 13 days of refrigerated storage (4°C) under modified atmosphere. Reuterin treatments were compared with washing with tap water or chlorinated water (250 mg/L), and with unwashed produce.

## Materials and Methods

### Bacterial Strain and Reuterin Stock Production

*Lactobacillus reuteri* DSM 20016T was obtained from the DSM strain collection (Leibniz Institute DSMZ-German Collection of Microorganisms and Cell Cultures, Braunschweig, Germany) and used for the production of reuterin using a two-step process, as described previously (Stevens et al., 2013). *L. reuteri* was routinely cultivated in Man, Rogosa and Sharpe medium (MRS, Biolife, Milan, Italy) at 37°C. Briefly, *L. reuteri* was grown overnight at 37°C in 10 mL MRS broth. One percentage (1%) of the overnight culture was added to filter sterilized MRS supplemented with 20 mM of glycerol (Sigma-Aldrich, Buchs, Switzerland) and incubated to an OD_600_ of approximately 8.0, representing the early stationary growth phase. To obtain cells with comparable metabolic activity among the trials, 10 mM of glucose and 20 mM of glycerol (final concentrations) were added to this early stationary phase culture and cells were reactivated for 30 min at 37°C. Subsequently, cells were harvested (4,000 × g, 10 min, room temperature) and re-suspended in sterile 600 mM glycerol solution. Conversion of glycerol to reuterin was conducted at 25°C for 3 h. Reuterin-containing supernatant was recovered by centrifugation (12,000 × *g*, 5 min, 4°C) and sterile filtered (0.2 μm). Several batches of reuterin were produced over a 10 day period, pooled together, and stored at 4°C for the washing trails. The freshly produced reuterin stock solution contained 433 mM 3-HPA and 6 mM acrolein. The same reuterin stock was used for all four washing trials, which were carried out over a total period of 7 months.

### HPLC-RI and IC-PAD Analysis of Reuterin Solutions

Glycerol, 1,3-PD, 3-HPA concentrations were determined by HPLC with refractive index detector (Hitachi LaChrome, Merck, Dietikon, Switzerland) on an Aminex HPX-87H column (300 mm × 7.8 mm, Bio Rad, Reinach, Switzerland) as described previously (Cleusix et al., 2008). Solutions for HPLC analysis were diluted in phosphate buffer (20 mM, pH 4.0) as previously recommended by Engels et al. (2016). Sulfuric acid (10 mM) was used as eluent and isocratic conditions were applied at a flow rate of 0.6 mL/min for 30 min at 40°C. The injection volume was 40 μL. Quantification was performed with external standards of glycerol and 1,3-PD (Sigma-Aldrich GmbH, Buchs, Switzerland). Purification of 3-HPA used as standard was done according to Vollenweider et al. (2003). HPA was diluted with distilled water to about 10 M solution, which was stable for at least 6 months during storage at 4°C.

Acrolein concentration was determined using IC-PAD as previously described (Engels et al., 2016). Hydroquinone (2%) was added to the samples to stabilize acrolein (Kächele et al., 2014). To minimize acrolein evaporation, airtight 0.7 ml PP Crimp/Snap LC vials (BGB Analytik, Boeckten, Switzerland) were used for analysis. Briefly, IC-PAD was performed on a Thermo Scientific (Reinach, Switzerland) ICS-5000+ system equipped with a quaternary gradient pump, a thermostated autosampler and an electrochemical detector with a cell containing a Ag/AgCl reference electrode and a disposable thin-film platinum working electrode tempered at 25°C. Analytes were separated with a Thermo Scientific IonPac ICE-AS1 4 × 250 mm ion-exclusion column with a guard column, operated at 30°C. The solvent system was isocratic 0.1 M methanesulfonic acid at 0.2 mL/min for 36 min. The injection volume was 10 μL. Electrochemical data were obtained after modification and optimization of the triple-potential waveform consisting of regeneration/detection, oxidation and reduction potentials. Commercial pure acrolein (>99%, stabilized with 0.2% hydroquinone) was purchased from Sigma-Aldrich GmbH (Buchs, Switzerland) and used as external standard. Acrolein is a volatile and toxic compound, hence, all safety measures were observed.

### Preparation of Lettuce Washing Solutions

Three different washing solutions (i) sodium hypochloride (NaOCl), (ii) enhanced reuterin (ER) and (iii) crude reuterin (CR) were prepared using autoclaved tap water. Sterile tap water washed (TW) and not washed (NW) lettuce were included as controls. To prepare NaOCl solution (2.5 L), liquid sodium hypochloride (Sigma-Aldrich) was added to autoclaved tap water to a final concentration of 250 mg/L free chlorine. On the day of washing, 2.5 L of CR washing solution was freshly prepared by mixing 580 mL reuterin stock to autoclaved tap water to achieve a final concentration of 100 mM 3-HPA. This solution (CR) was then cooled down on ice to 4°C and used immediately. For preparing ER, 2.5 L of CR solution was prepared as presented above and incubated for 16 h at 50°C in a dry oven (Cleanroom drying oven UF750plus, Memmert GmbH + Co. KG, Schwabach, Germany) to shift the reuterin equilibrium from 3-HPA to acrolein. The ER solution was cooled down on ice to 4°C and immediately used for washing.

### Preparation and Processing of Romaine Lettuce Samples

**FIGURE 1:**
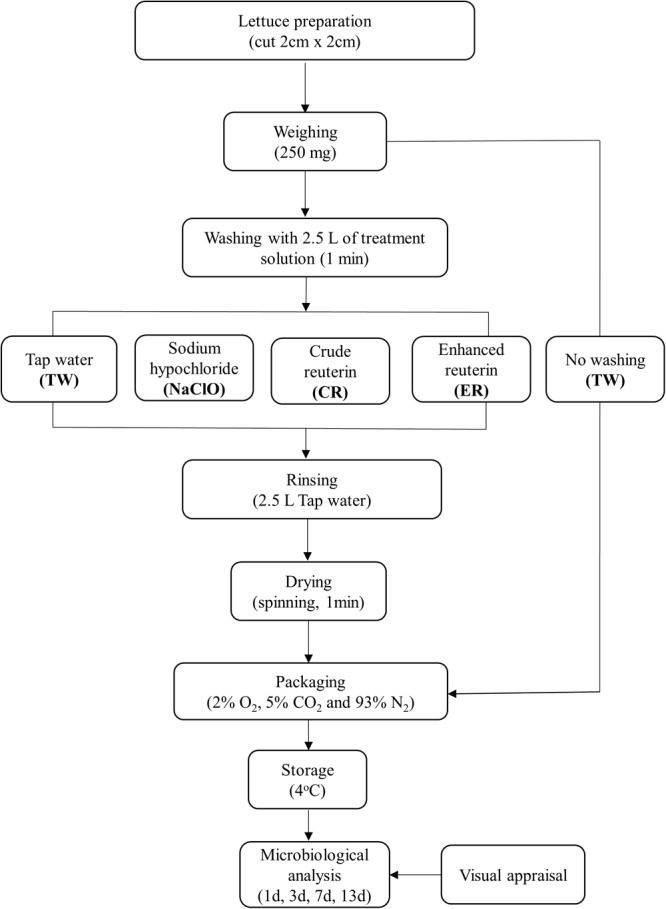
Experimental set up of the washing trials of minimally-processed (MP) lettuce using different treatments.

Romaine lettuce (*Lactuca sativa*) from Switzerland (Zürich area) which had not been prewashed was purchased from a local retailer on the same day of arrival from the grower. Trial 1, 2, and 3 were performed at intervals of 14 days with lettuce obtained during the winter season of 2016, whereas trial 4 was performed with lettuce obtained in the spring season of 2017 (7 months after trial 1). The lettuce heads were transported under cold conditions to ETH Zürich and immediately processed and washed in conditions simulating continuous MP process in industry, according to the flow diagram described in **Figure 1**. Soiled, damaged outer leaves, and stem were removed and leaves were cut (approximately 2 × 2 cm) using disinfected knives.

For each treatment, 250 g of lettuce were washed by hand with gentle mixing in a 5 L beaker containing 2.5 L of solution (1:10 w/v) for 1 min. Following the washing process, the products were placed in a sieve for 30 s to allow the washing water to drain off. The products were then rinsed with 2.5 L of autoclaved tap water to remove residue treatment on the product and a salad spinner was used to manually centrifuge the washed lettuce. Lettuce was packed into polyethylene sterile bags (18 × 12 cm, 50 g each) under modified atmosphere (2 ± 1% O_2_, 5 ± 1% CO_2_, and 93 ± 1% N_2_), as previously described by Pereira et al. (2014). For packing, a gas exchange device with a vacuum packaging machine (Multivac C200, Sepp Haggenmüller GmbH & Co. KG, Wolfertschwenden, Germany) and a mixing station (Gas mixer KM 100-3M, Witt-Gasetechnik GmbH & Co. KG, Written, Germany) were used.

Five milliliter of the washing solution were collected before and after lettuce washing to measure the concentrations of glycerol, 3-HPA, 1, 3-PD and acrolein, and the pH (Metrohm 780 pH Meter, Metrohm AG, Herisau, Switzerland).

### Bacteria, Yeasts and Molds Counts During Storage

The population of aero-tolerant microbes was enumerated in duplicate after 1, 3, 7, and 13 days of storage at 4°C. At each sampling point, the content of a package (50 g) was transferred into a stomacher bag (Stomacher^®^ 400 Classic Bags, Seward, West Sussex, United Kingdom), combined with 200 mL of 0.1% peptone and macerated in a stomacher (BagMixer^®^ 400 P, Interscience, Saint Nom, France) at high speed for 5 min. Serial dilutions were made in 0.1% peptone and surface plated (0.1 mL) in duplicate onto selective and non-selective media.

Total aerobic mesophilic bacteria were enumerated in duplicate on Luria-Bertani (LB) agar (Becton Dickinson, Allschwil, Switzerland), and *Enterobacteriaceae* were grown using ENDO agar (Sigma-Aldrich, Buchs, Switzerland). LB and ENDO agar were incubated at 37°C for 48 h. Yeasts and molds were enumerated using Yeast extract Glucose Chloramphenicol (YGC) agar (Merck KGaA, Darmstadt, Germany) further supplemented with 10 μg m/L chloramphenicol after autoclaving, and the plates were incubated at 30°C for 48 h.

### Acrolein Quantification in Lettuce Washed With Reuterin

To estimate the residual acrolein concentration in reuterin washed lettuce, 2 mL of macerated lettuce prepared above was centrifuged at 4°C at 5000 × *g* for 5 min. The supernatant was passed through a 0.45 μm filter (Infochroma AG, Switzerland), and acrolein concentration was determined using IC-PAD.

### Visual Evaluation of Lettuce Leaves

The appearance of fresh-cut lettuce was assessed visually after 1, 3, 7, and 13 days after treatment. The focus was on discoloration, browning of cut edges and appearance of brown spots on the leaves.

### Data Analysis

The experiments were carried out with four blocks using the same reuterin stock, with each block including washing treatments with TW, CR, ER, and NaClO compare to un-washed lettuce. Plate counts were performed in duplicate, and mean data were reported as colony-forming units per gram lettuce (CFU/g). The decimal reduction of a washing treatment was calculated at each time point by the difference between the treatment and unwashed lettuce count. To assess the dose dependent effects of acrolein on cell counts, a regression analysis was used with CR and ER data after 1 and 13 days of storage, using SigmaPlot 12.5 (Systat Software, San Jose, CA, United States).

## Results

### Fate of Reuterin Components During Washing

A single stock of reuterin was produced for the four trials carried out over a total period of 7 months. It was observed that the composition of CR prepared from the stock solution changed over time, with a steady decrease of 3-HPA concentration, from 98.5 in trial 1 to 87.1 mM in trial 4. However, the acrolein concentration of 1.8 ± 0.2 mM in the CR stock did not change with time (**Table 1**). The concentrations of 3-HPA and acrolein in the solution collected after washing the lettuce were slightly lower than before washing for CR treatments. Between 0.6–1.3 mM 3-HPA and 0.1–0.7 mM acrolein were not recovered in the CR washing solution after washing.

**Table 1:** 3-HPA and acrolein concentrations in washing solution quantified by HPLC and IC-PAD. Acrolein loss during washing was estimated by the difference of concentration in the solution before and after washing.

Washing solution	Washing step	Trial 1 [mM]		Trial 2 [mM]		Trial 3 [mM]		Trial 4 [mM]	
		3-HPA	Acrolein	3-HPA	Acrolein	3-HPA	Acrolein	3-HPA	Acrolein
Crude reuterin, CR	Before washing	98.5	1.5	95.5	1.9	93.9	1.7	87.1	1.9
	After washing	97.2	1.4	94.8	1.7	92.9	1.0	86.5	1.8
	Loss	1.3	0.1	0.7	0.2	1.0	0.7	0.6	0.1
Enhanced reuterin, ER	Before washing	92.5	12.1	86.7	21.9	85.7	20.9	75.8	7.2
	After washing	90.7	11.5	85.2	20.3	83.2	20.2	71.8	6.4
	Loss	1.8	0.6	1.5	1.6	2.5	0.7	4.0	0.8

Acrolein was recently shown to be the main component for antimicrobial activity of reuterin (Engels et al., 2016). Therefore we incubated the CR reuterin solutions at 50°C for 16 h to produce ER solutions containing an increased acrolein concentration. The 3-HPA concentration in ER decreased from trial 1 (92.5 mM) to trial 4 (75.8 mM), consistent with the decreased concentration in CR observed over time. The acrolein titres in ER solutions for the four trials were in the range of 7.2–21.9 mM, which corresponded to between 4- and 12-fold increase compare to the corresponding CR solutions, but with no time effect. The variation in acrolein synthesis in ER solution was due to the oven not able to control the set temperature during incubation. In case where 7.2 and 12.1 mM acrolein was obtained, the temperature of the oven as measured by external thermometer was 5°C less than the set temperature. Between 2–4 mM 3-HPA and 0.6–1.6 mM acrolein were not recovered in the ER washing solution after washing (**Table 1**).

### Antimicrobial Effect of Washing Solutions

**FIGURE 2:**
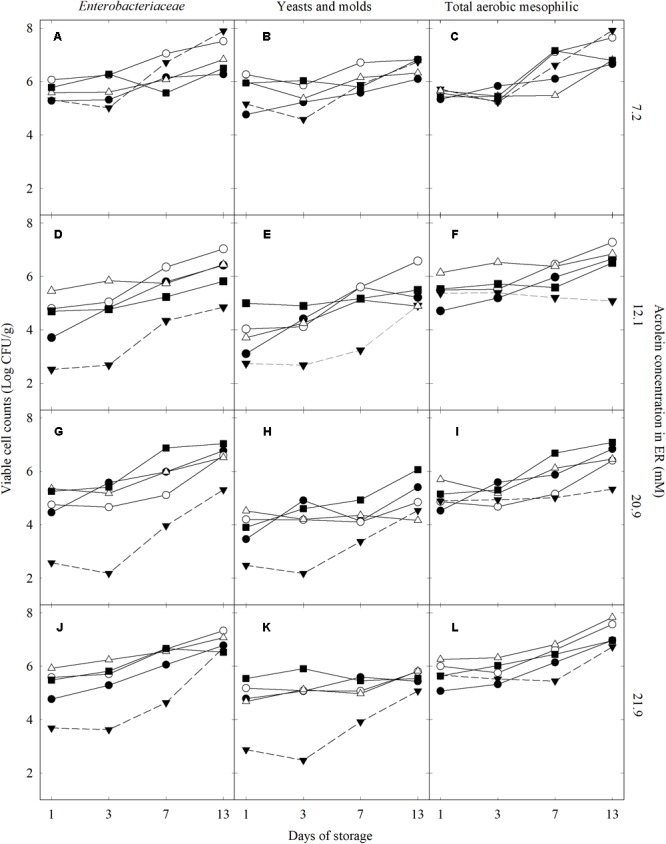
*Enterobacteriaceae*, yeasts and molds, and total aerobic mesophilic viable cell counts in lettuce washed with different treatments and stored for 13 days at 4°C under protective atmosphere: crude (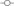) and enhanced (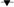) reuterin, NaClO (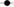), water (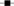), and unwashed (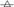). Data was grouped based on the acrolein concentration in the ER washing solution: 7.2 mM (**A–C**; trial 4), 12.1 mM (**D–F;** trial 1), 20.9 mM (**G–I;** trial 3), and 21.9 mM (**J–L;** trial 2).

We investigated the impact of different washing solutions (CR, ER, NaClO, and TW) on the viable cell counts of *Enterobacteriaceae*, yeasts and molds, and total aerobic mesophilic bacteria of lettuce after washing and during storage for 13 days at 4°C (**Figure 2**). The effectiveness of treatments was also expressed by the decimal reduction of the counts for a treatment at a defined time point compare to the unwashed lettuce NW (Supplementary Figure 1).

Fresh-cut lettuce washed with 250 mg/L of NaClO showed a decimal reduction of the *Enterobacteriaceae* population between 0.3 and 1.8 log CFU/g compare to NW (5.6 ± 0.2 log CFU/g, *n* = 4) after day 1 of storage. The counts of *Enterobacteriaceae* steadily increased with storage time to reach similar levels to the unwashed lettuce at day 13 (6.7 ± 0.1 and 6.7 ± 0.3, respectively). Washing with TW, CR, and ER with 7.2 mM acrolein (trial 4) resulted in less than 1 log CFU/g decimal reduction after 1 day compare to NW, followed by regrowth of *Enterobacteriaceae* on lettuce during storage to reach similar levels as for NW after 13 days. In contrast, washing with ER containing 12.1 (trial 1), 20.9 (trial 3), or 21.9 mM acrolein (trial 2) strongly reduced the initial counts of *Enterobacteriaceae* between 2.1 and 2.8 log CFU/g, after the first day of treatment compare to NW. The decimal reductions induced by ER treatments (12.1 mM or more acrolein) were maintained until day 7 (between 1.4 and 2.0 log CFU/g), while a pronounced regrowth of *Enterobacteriaceae* occurred after day 3. At the end of storage, *Enterobacteriaceae* counts in active ER treatments were similar to the other washing treatments and NW lettuce (**Figures 2D,G,J**).

The yeasts and molds counts (NW, 4.7 ± 0.9 log CFU/g, *n* = 4) was reduced by less than 1 log unit after washing with TW, CR, and ER with 7.2 mM acrolein after 1 day of storage compared to NW. Washing with NaClO resulted in a 0.6–1.2 log CFU/g decimal reduction of yeasts and molds after the first day of storage. ER washing treatments containing 12.1, 20.9, and 21.9 mM acrolein reduced the initial counts of yeasts and moulds between 1.3 and 2.0 log CFU/g after day 1 of treatment. The ER inhibition effect was maintained until day 3. At day 7, while yeasts and molds started to regrow, the decimal reduction remained between 1.0 and 1.8 log CFU/g compared to unwashed lettuce. At day 13, the yeasts and molds counts on lettuce washed with ER containing acrolein 12.1 mM and above were similar to unwashed lettuce (5.3 ± 0.9 log CFU/g). For NaClO, TW, and CR treatments, growth of yeasts and molds were already noticeable at day 3, and at day 13, the counts of yeasts and molds on the stored lettuce were 5.5 ± 0.4, 5.8 ± 0.3, and 6.0 ± 0.4 log CFU/g (*n* = 4), respectively.

The total aerobic mesophilic bacteria counts were reduced between 0.3 and 1.4 log CFU/g on day 1 after NaClO washing compare to NW lettuce (5.9 ± 0.3 log CFU/g, *n* = 4). TW, CR, and ER treatments resulted in a limited reduction of total aerobic mesophilic bacteria, less than 1 log after 1 day of storage compare to NW. At day 7, washing with ER containing 12.1 mM or more acrolein still reduced the growth total aerobic mesophilic bacteria between 1.1 and 1.4 log CFU/g compared to unwashed lettuce. This effect was maintained until the end of storage in trail 2 (12.1 mM) and 3 (20.9 mM) (**Figures 2F,I**). The counts of total aerobic mesophilic bacteria of TW, CR, and NaClO were similar to unwashed lettuce at the end of the 13 day storage (**Figures 2F,I,L**), except in trial 4 (**Figure 2C**).

### Relationship Between Acrolein Concentration and Antimicrobial Effect

**FIGURE 3:**
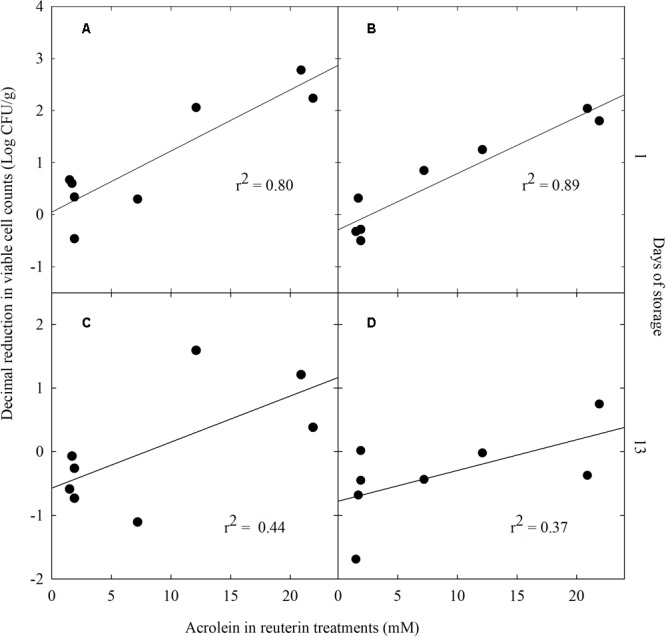
Linear regression analysis of decimal reduction of *Enterobacteriaceae* **(A,C)** and yeasts and molds **(B,D)** viable cell counts versus acrolein concentration in crude reuterin (CR) and enhanced reuterin (ER) treatments after washing and storage for day 1 **(A,B)** and day 13 **(C,D)**.

A regression analysis was applied to test the dose dependent effect of acrolein on the decimal reduction of *Enterobacteriaceae* and yeasts and molds which were most affected compare to NW lettuce. A significant linear dependency was calculated between the decimal reduction of *Enterobacteriaceae* (*r*^2^ = 0.80, *P* < 0.05) and of yeasts and molds (*r*^2^ = 0.89; *P* < 0.05) after 1 day and acrolein concentration of the washing treatment (**Figures 3A,B**, respectively). On day 13 of storage there was no significant correlation between acrolein concentration and decimal reduction of *Enterobacteriaceae* (*r*^2^ = 0.40, *P* > 0.05) and of yeasts and molds (*r*^2^ = 0.37; *P* > 0.05) (**Figures 3C,D**, respectively).

### Visual Appearance of Washed Lettuce During Storage

**FIGURE 4:**
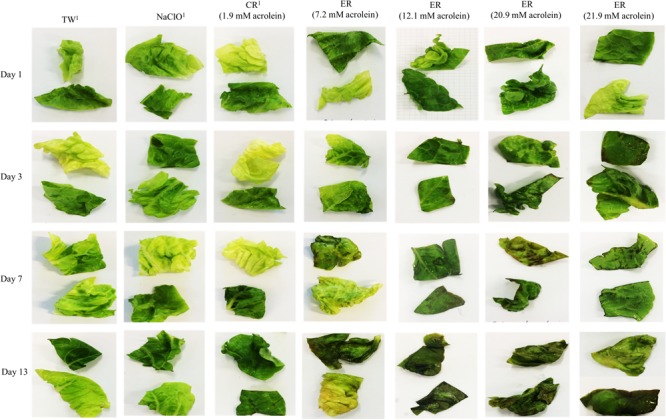
Visual evaluation of lettuce after washing with different treatments and during 13 days of storage under modified atmosphere. Washed with water (TW), NaClO, ER, and CR. ^1^Images obtained from trial 2. Selected images of damaged leaves illustrating spoilage of different treatments. The image may not be representative of the entire lettuce.

Differences in the visual appearance of lettuce washed with ER solutions were observed starting on day 3 for lettuce washed with ER containing 20.9 and 21.9 mM acrolein. At 7-days storage at 4°C, marked discoloration were observed for lettuce washed with reuterin treatment containing 7.2, 12.1, 20.9, and 21.9 mM acrolein. The effect was slightly more pronounced with increasing acrolein concentration (**Figure 4**). Lettuce washed with NaClO, TW, and CR showed similar visual quality in all four trials, and hence, we present images from trial 2 to represent all four trials.

## Discussion

### Acrolein Is the Main Antimicrobial Compound in Reuterin Washed Lettuce

The mechanistic basis of reuterin’s antimicrobial activity has been proposed to be an imbalance in cellular redox status resulting from reactions of 3-HPA with free thiol groups, causing the depletion of glutathione and modification of proteins, including functional enzymes within the cells (Schaefer et al., 2010; Vollenweider et al., 2010). We recently showed that acrolein is the compound of the reuterin system responsible for antimicrobial activity on indicator strains in simple buffered media (Engels et al., 2016). Using lettuce as a model vegetable, we confirmed the activity of acrolein as the main antimicrobial of the reuterin system in a complex food matrix, with a dose-dependent activity on *Enterobacteriaceae* and on yeasts and molds.

The antimicrobial potential of CR as a food biopreservative was previously investigated against different food-borne pathogens like *S. aureus, L. monocytogenes, E. coli* O157:H7, or *S. enteritidis* in different food matrices, including cold-smoked salmon (Montiel et al., 2014, 2016), milk and dairy products (Arqués et al., 2004, 2008a,b). However, no significant antimicrobial effect was observed when CR was applied at concentrations ranging from 2 to 8 AU/g or mL in food stored at refrigeration temperatures (4–10°C). Interestingly, bactericidal activity of reuterin (8 AU/ml) against *L. monocytogenes* and *S. aureus* in milk was reported after 24 h of incubation at 37°C (Arqués et al., 2004). In line with the observation that acrolein is the main antimicrobial component of the reuterin system (Engels et al., 2016), it may be speculated that incubation of milk at 37°C promoted the continuous formation of acrolein from 3-HPA leading to the bactericidal activity of reuterin against *L. monocytogenes* and *S. aureus.* In our study, a simple heat treatment of CR at 50°C for 16 h was used to cause a 4–12 folds increase in acrolein concentration in enhanced reuterin preparation.

Most investigations carried out with reuterin in food systems did not provide information on purity of reuterin, and the exact concentrations of 3-HPA and acrolein were not disclosed. Instead reuterin activity was determined using arbitrary activity tests which does not allow comparing results between different studies. Therefore the exact composition of reuterin, and more specifically the concentration of acrolein in the conditions of the test should be established for rigorous testing of reuterin as a disinfectant or preservative in food.

### Acrolein Inhibits Enterobacteriaceae, Yeasts and Molds in Washed Lettuce

The sanitation of vegetable produce is intended to reduce the natural microbial load to increase shelf life and ensure safety of raw consumed vegetables. The effect of decontamination solutions used in MP vegetable produce on the natural lettuce microbiota (total aerobic mesophilic count) was usually reported smaller than that obtained with artificially inoculated bacteria (Oliveira et al., 2015). Our results showed that the population of initial total aerobic mesophilic bacteria was less affected by all the treatments applied than *Enterobacteriaceae*, and yeasts and molds. This may be partly due to biofilm formation by native lettuce microbiota, which may result in a protective effect from the action of antimicrobial compounds (Hunter et al., 2010; Vorholt, 2012). Pathogenic bacteria from the *Enterobacteriaceae* family such as *Salmonella, Shigella*, and *E. coli* O157:H7 mostly from facal origin have been involved in outbreaks of foodborne diseases associated with the consumption of fresh-cut produce (Ramos et al., 2013). Therefore, the counts of *Enterobacteriaceae* are of special importance to assess the efficacy of a microbial-reducing process in respect of food safety. The application of enhanced reuterin washing with acrolein concentration of 12.1 mM and higher on fresh-cut lettuce resulted in a strong reduction of *Enterobacteriaceae* for at least 7 days compared to unwashed lettuce and all the other tested conditions, including CR, ER with 7.2 mM acrolein, TW and NaClO. Our results are in agreement with previous finding suggesting that Gram-negative bacteria are more sensitive to reuterin than Gram-positive bacteria (Chung et al., 1989; El-Ziney et al., 1999; Arqués et al., 2004; Stevens et al., 2010).

The role of yeasts and molds in fresh-cut vegetables spoilage and mycotoxin production was previously reported by Tournas (2005). In our study, between 1.3 and 2.0 log unit reductions in the count of yeasts and molds were observed after 1 day storage of lettuce washed with ER treatments containing between 12.1 and 21.9 mM acrolein. The MIC of reuterin on several yeasts and filamentous fungi were previously reported between 0.15 and 3 mM (Chung et al., 1989; Stevens et al., 2010). Sanitizing agents of fresh-cut produce may be considerably more effective against yeasts and molds than against aerobic mesophilic microbiota, because the later include several Gram-positive spore-forming bacteria of the *Bacillus* genus, known to be more resistant to chemical sanitizing agents (Brackett and Splittstoesser, 2001).

After an initial reduction after washing with ER, *Enterobacteriaceae*, and yeasts and molds counts increased steadily, eventually reaching equal levels of the other treatments and the unwashed produce at the end of the 13 day storage period. Several authors have demonstrated that the washing steps commonly used in MP produce were only bacteriostatic as microbial populations recover to prewashed numbers at the end of storage (Gómez-López et al., 2008; Tirpanalan et al., 2011). This also emphasizes that a decontamination process may not be aimed at increasing the shelf-life of the product but rather enhance its safety during the normal storage life.

Many studies have identified that chlorine is the most popular disinfection method for fresh produce (Shen et al., 2012; Van Haute et al., 2013). Sodium hypochlorite (NaClO) is the source of chlorine commonly used by the food industry for sanitizing both products and equipments (Gil et al., 2009). For efficacy and stability, the pH should be kept in the range of 6.5–7.5 (Suslow, 1997). Using 50–200 ppm of hypochloride and 1–2 min contact time at this pH results in a maximum of 1–2 log reduction of the initial total aerobic microbes in many commodities (Parish et al., 2003; Gonzalez et al., 2004; Allende et al., 2008). Pezzuto et al. (2016) recently report that NaClO (200 mg/L) was able to reduce *Salmonella* counts in leafy green vegetable by 2 log units, but only in the case of a high initial contamination (7 log CFU/g). In our study the NaClO washing solution at uncontrolled pH of approximately 9.0 led to a reduction between 0.9 and 1.8 log of *Enterobacteriaceae*, total aerobic mesophilic bacteria and yeasts and molds counts after washing, except for trial 4 which showed no effect of chlorine. The lack of efficiency could be associated with difference in microbiota composition of the lettuce used in trial 4, evident by high numbers of initial counts of yeasts and molds in trial 4, compare to other trials.

Since the ban of the use of chlorine for decontamination of fresh produce in some European countries, most fresh-cut producers currently use tap water for washing. Our results indicate that washing fresh-cut produce with tap water alone was not sufficient to significantly reduce the initial microbial load on the fresh-cut lettuce (0.3–0.6 log reduction). Similar results were obtained by Gonzalez et al. (2004) who found that washing shredded carrots with water reduced total aerobic microbial counts by only 0.3–0.4 log units, regardless of the water quality used. Additionally, Luo (2007) did not find differences in the aerobic mesophilic growth of Romaine lettuce after washing with potable tap water.

In our study, washing fresh-cut produce with reuterin treatments containing acrolein in the range between 12.1 and 21.9 mM resulted in a reduction of *Enterobacteriaceae*, yeasts and molds in the range from 1.3 to 2.8 log units, well above the effects observed with chlorine and tap water washing.

### Reuterin Washing Causes No Detectable Residual Acrolein in the Produce but Lead to Discoloration of Fresh-Cut Lettuce During Storage

The fate of reuterin and its degradation product is of importance for predicting the safety of the produce after washing (Fernández-Cruz et al., 2016). The main antimicrobial component of the reuterin system, acrolein, is highly volatile, colorless, and may be present in many food, sometimes at high levels of more than 4.0 ng/g such as in potato chips fried in corn oil (Watzek et al., 2012). Acrolein is considered a highly cytotoxic compound after a single exposure, hence a tolerable daily intake of 0.75 mg/kg body weight/day was suggested (Abraham et al., 2011; Fernández-Cruz et al., 2016; Zhang et al., 2018). In our study, between 0.6–1.6 mM and 0.1–0.7 mM of acrolein was lost during washing with ER and CR, respectively. We therefore tested acrolein in the rinsing water and in washed lettuce using the high sensitive IC-PAD method developed by Engels et al. (2016). We did not detect acrolein in the macerated lettuce and rinsing water after washing with ER solutions, with the detection limit of the method of 4.4 μM. Because acrolein is a volatile compound, we speculate that part of unrecovered acrolein in the wash solution may have been lost by volatization. It was previously reported that because of its volatility, acrolein was not observed after 1 day in lettuce grown on soils irrigated with MAGNACIDE@ H Herbicide which contains at least 92% acrolein (Nordone et al., 1997). Aside volatilization and reaction with microbes and plant components, reversible, first-order hydration of acrolein to 3-HPA which is enhanced at low temperatures may be a significant pathway for the elimination (Engels et al., 2016). However, little is known about reactions and degradation products of acrolein which is a very reactive component (Engels et al., 2016; Zhang et al., 2017, 2018). Taken together, the absence of detectable acrolein in the treated lettuce suggest safety of reuterin washing at effective acrolein concentrations, but additional testing may be needed to identify potential by-products of acrolein.

Quality preservation is, after safety, the most important attribute of minimally processed vegetables, since purchasing decisions often depends on consumers satisfaction in terms of visual, textural and flavor quality of the product (Allende et al., 2008; Barrett et al., 2010). In this study, we observed increased brown discoloration of the fresh-cut lettuce after washing with ER containing acrolein concentrations in the range from 7.2 to 21.9 mM and during subsequent storage. The dark green color of the cut-lettuce started to decrease from day 3 and continued to decline throughout the remaining storage period. Interestingly, the point of discoloration of lettuce after washing with ER corresponds to regrowth of *Enterobacteriaceae* and yeast and molds. We speculate that these microbes were able to multiply faster once the tissue has been damage. Unwashed and NaClO washed samples maintained fresh appearance during 13 day storage. This is in agreement with previous studies where washing fresh-cut lettuce with chlorinated water reduced browning (Baur et al., 2004). The major enzyme controlling oxidative discolouration of cut lettuce has been reported to be polyphenol oxidase (PPO) (Hunter et al., 2017). Acrolein induces oxidative stress in cells, due to the oxidation of phenolic compounds (Schaefer et al., 2010). We speculate that this deterioration of produce washed with enhanced reuterin is due to acrolein oxidation of the phenolic compounds in the cut lettuce. Further studies are required to understand the phenomenon for the increase browning on lettuce washed with enhance reuterin on a molecular level.

## Conclusion

Our study shows that in food system as for laboratory test conditions, acrolein is the main antimicrobial component of the reuterin system because only the application of ER containing between 12.1 and 21.9 mM acrolein reduced initial counts of *Enterobacteriacea*, yeasts and molds on fresh-cut lettuce. From the microbiological point of view, the use of reuterin with enhanced acrolein ensured the acceptability of modified atmosphere packaged lettuce for up to 7 days of storage at 4°C and reduced the risks associated with putative pathogenic genera’ of the *Enterobacteriaceae* family. However, at effective acrolein concentration, reuterin may not be used to extend the shelf-life of fresh-cut lettuce, due to chemical reactions changing the visual appearance. The application of reuterin for the decontamination and biopreservation of other vegetable less sensitive to oxidative stresses than lettuce, such as carrot, apples, beets, and radish, should be tested. Furthermore the reuse and recycling ER washing solutions containing high levels of acrolein should be investigated to limit environmental impact.

## Author Contributions

PA, AG, CS, MJS, and CL designed the study. PA, AG, CS, and MS conducted the experiments and analyzed the data. PA, AG, and CL drafted the manuscript. All authors read and approved the final manuscript.

## Conflict of Interest Statement

The authors declare that the research was conducted in the absence of any commercial or financial relationships that could be construed as a potential conflict of interest.
