# Supplementary material for: Decontamination of Minimally-Processed Fresh Lettuce Using Reuterin Produced by Lactobacillus reuteri
Source: Front Microbiol. 2018 Jul 4;9:1421. doi: 10.3389/fmicb.2018.01421 (PMC6040215; doi:10.3389/fmicb.2018.01421)
Supplement: Supplementary file 1 [file Image_1.pdf]

Supplementary Material

Decontamination of minimally-processed fresh lettuce using reuterin produced by *Lactobacillus reuteri*

Paul Tetteh Asare, Anna Greppi, Martina Stettler, Clarissa Schwab, Marc J. A. Stevens, Christophe Lacroix*

*Correspondence: Christophe Lacroix: christophe.lacroix@hest.ethz.ch

1. Supplementary Figure

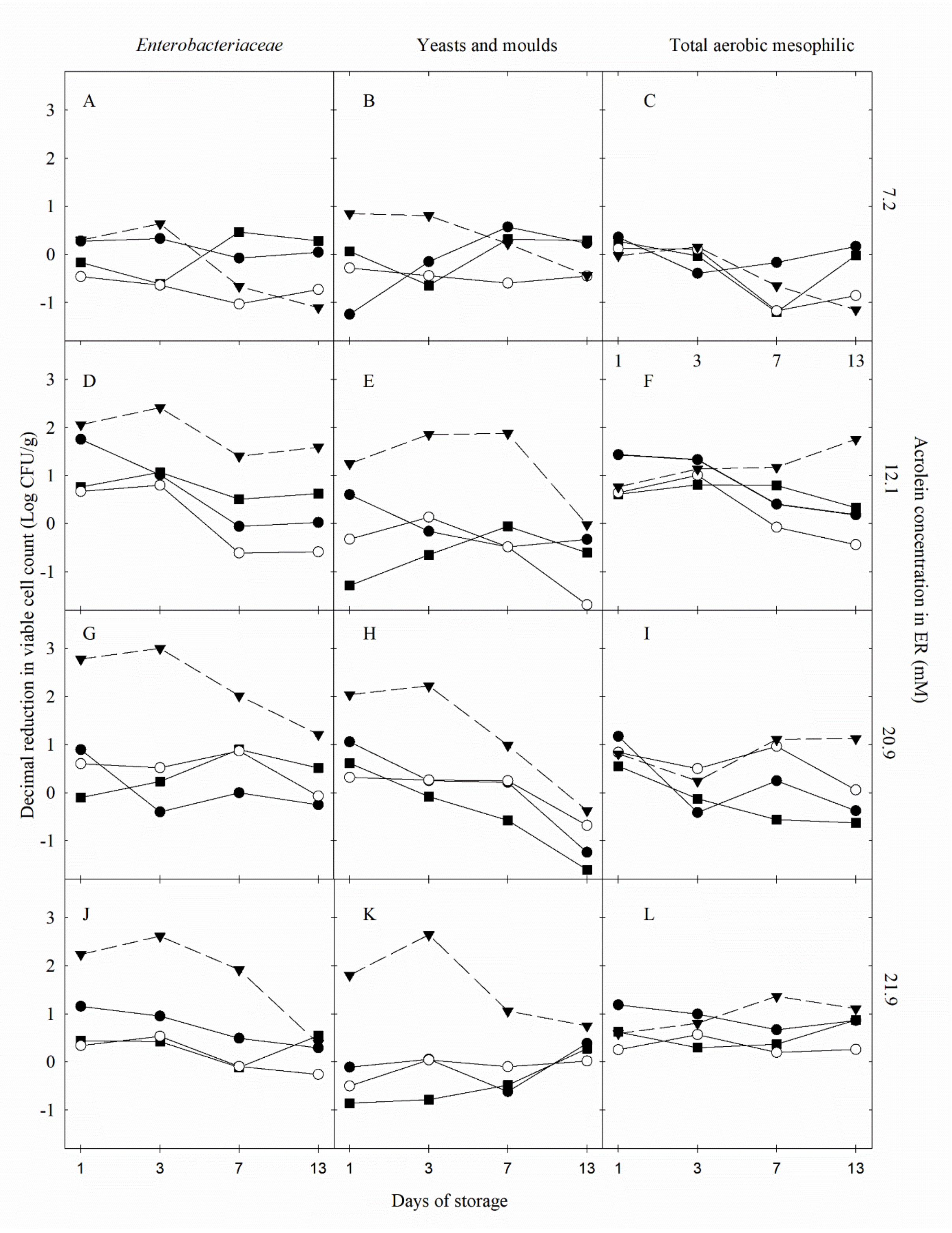

Supplementary Figure 1: Decimal reduction (log CFU/g) of *Enterobacteriaceae*, yeasts, moulds and total aerobic mesophilic on lettuce washed with different treatments and stored for 13 days at 4°C under protective atmosphere compared to unwashed lettuce: crude (-○-) and enhanced (-▼-) reuterin, chlorine (-●-), tap water (-■-). Data was grouped based on the acrolein concentration in the ER washing solution: 7.2 mM (**A, B, C**; trial 4), 12.1 mM (**D, E, F**; trial 1), 20.9 mM (**G, H, I**; trial 3) and 21.9 mM (**J, K, L**; trial 2)
